# Supplementary material for: Identification of risk genes for Alzheimer’s disease by gene embedding
Source: Cell Genom. 2022 Jul 26;2(9):100162. doi: 10.1016/j.xgen.2022.100162 (PMC9581494; doi:10.1016/j.xgen.2022.100162)
Supplement: Document S1. Figures S1–S7 [file mmc1.pdf]

Cell Genomics, Volume *2*

Supplemental information

Identification of risk genes for Alzheimer's disease by gene embedding

Yashwanth Lagisetty, Thomas Bourquard, Ismael Al-Ramahi, Carl Grant Mangleburg, Samantha Mota, Shirin Soleimani, Joshua M. Shulman, Juan Botas, Kwanghyuk Lee, and Olivier Lichtarge

Supplementary Information for
Identification of Risk Genes for Alzheimer's Disease by Gene Embedding.

Yashwanth Lagisetty[1,2], Thomas Bourquard[2], Ismael Al-Ramahi[2,3,4], Carl Grant Mangleburg[2], Samantha Mota[2], Shirin Soleimani[2], Joshua M. Shulman[2,3,4,5,6], Juan Botas[2,3,4], Kwanghyuk Lee[2], Olivier Lichtarge* [2,4,7]

* To whom correspondence should be addressed:
Olivier Lichtarge MD, PhD
Email: lichtarge@bcm.edu

Supplemental Figure Legends

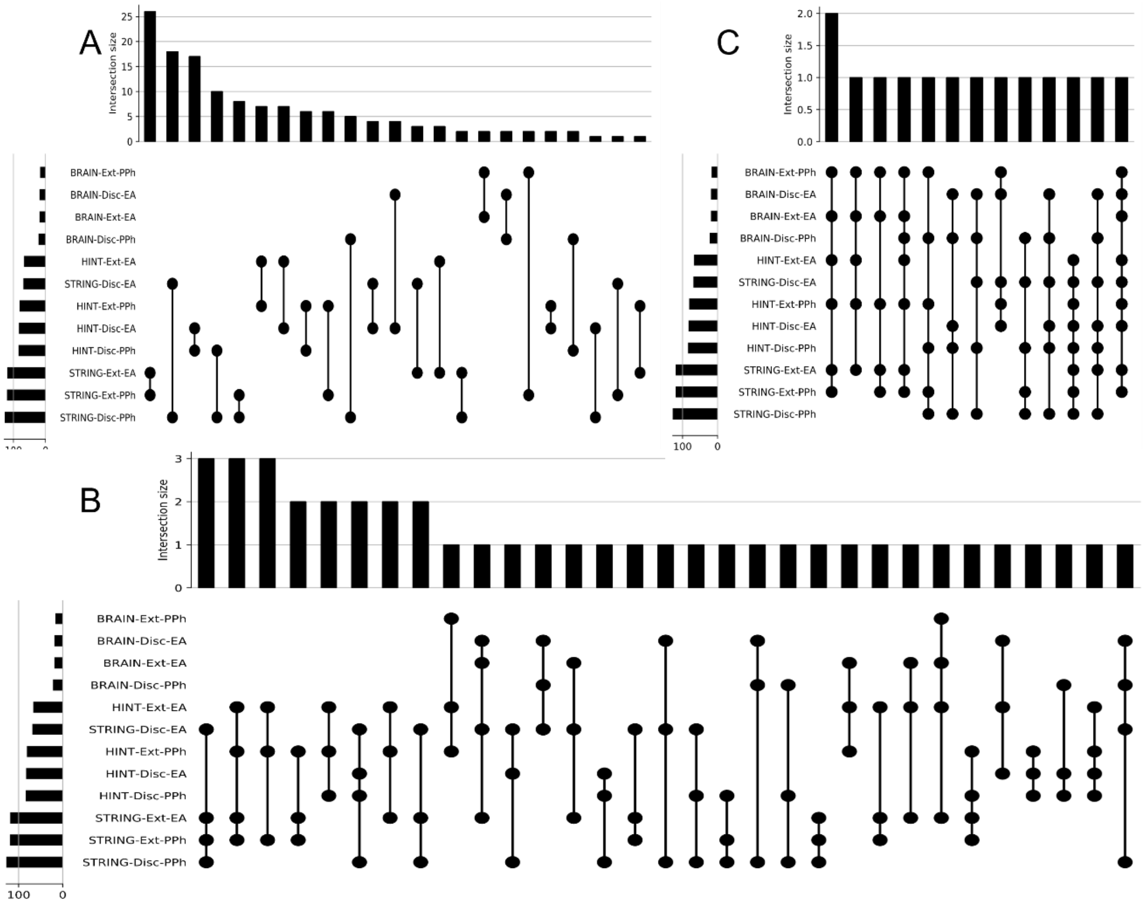

Figure S1. UpSet plot of combinatorial intersections between all 12 GeneEMBED experiments, Related to Figure 4. (A) pairwise intersections. (B) Intersections between 3-4 sets.

(C) Intersections between 5+ sets. Set of 143 unique genes from all intersections are used for 'high-confidence' gene set.

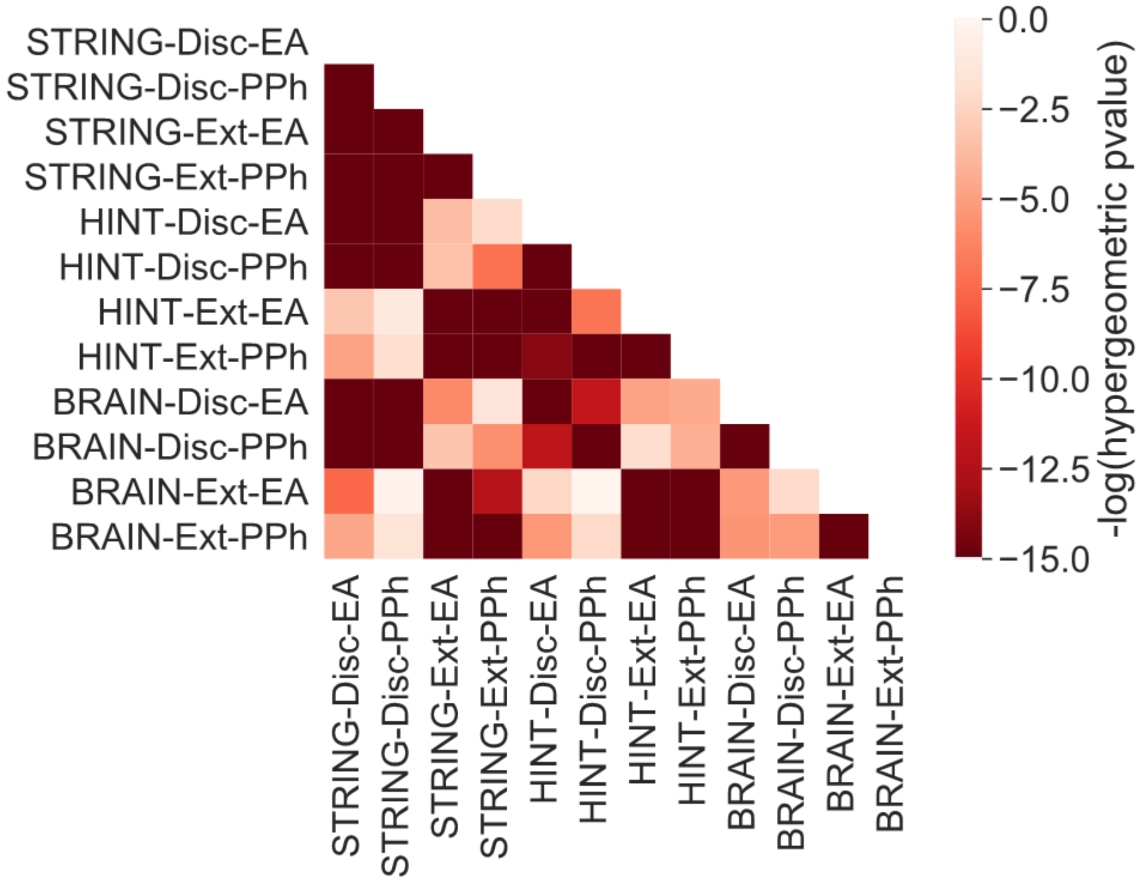

Figure S2. GeneEMBED candidates are consistently identified across various cohorts, networks, and VIS systems, Related to Figure 4. One-tailed hypergeometric overlap tests were done on every pairwise combination of cohort-network-VIS experiments. Among 66 independent pairwise tests, only 11 did not demonstrate statistically significant hypergeometric p-values ($p < 0.05$, $\log(p) < -2.99$).

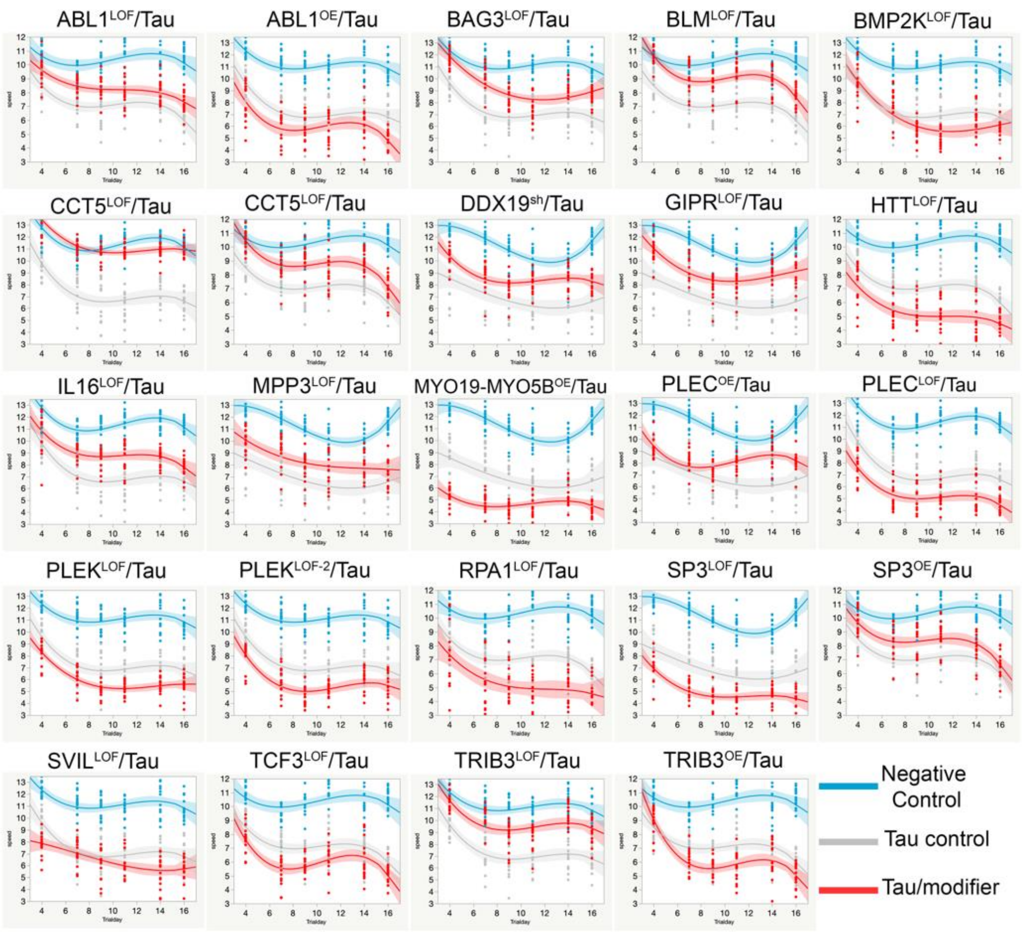

Figure S3. GeneEMBED candidates modulate tau-induced neuronal dysfunction, Related to Figure 4. Regressions representing average speed as a function of age in control fruit flies (blue) or flies expressing human wild type _Tau_ either alone (grey) or together with the above indicated modifiers (red) on the corresponding Drosophila homolog (see supplementary table 12 for genotype details). Charts show third degree polynomials and confidence intervals. All differential effects were statistically significant ($p<0.01$) following ANOVA analysis on Linear mixed models regression with fitted splines

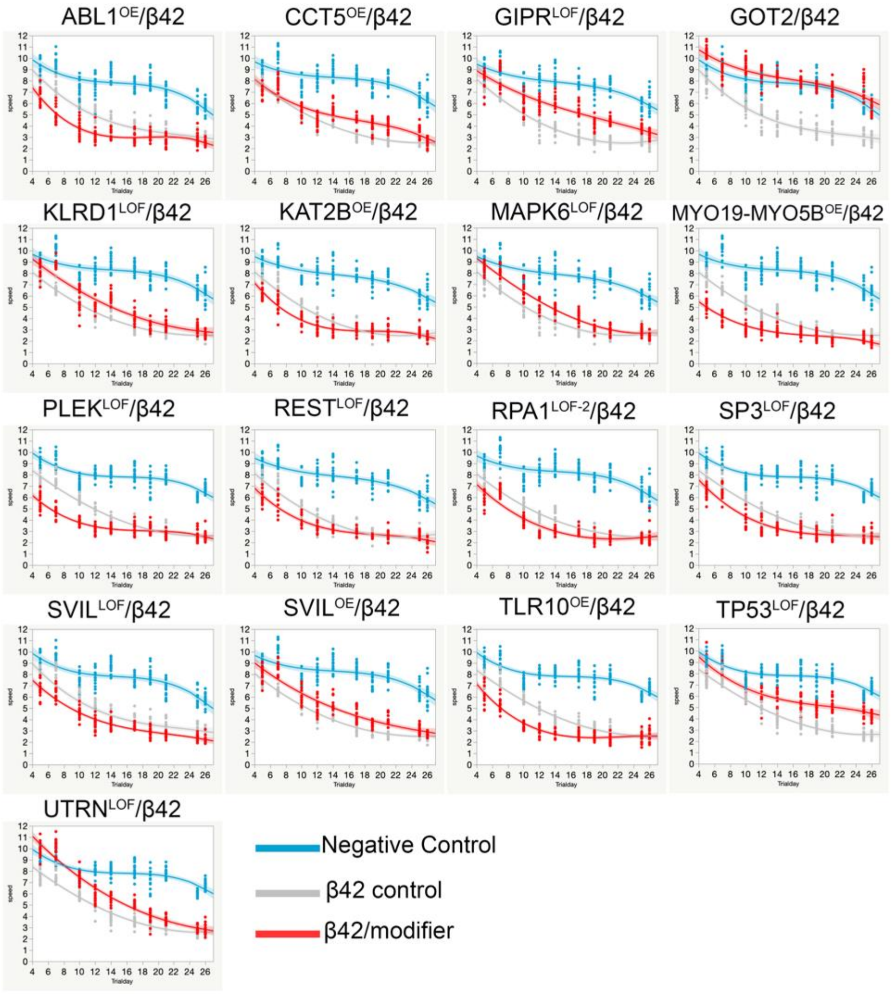

Figure S4. GeneEMBED candidates modulate β amyloid-induced neuronal dysfunction, Related to Figure 4. Regressions representing average speed as a function of age in control fruit flies (blue) or flies expressing human wild type *β amyloid* either alone (grey) or together with the above indicated modifiers (red) on the corresponding Drosophila homolog (see supplementary table 12 for genotype details). Charts show third degree polynomials and confidence intervals. All differential effects were statistically significant ($p<0.01$) following ANOVA analysis on Linear mixed models regression with fitted splines

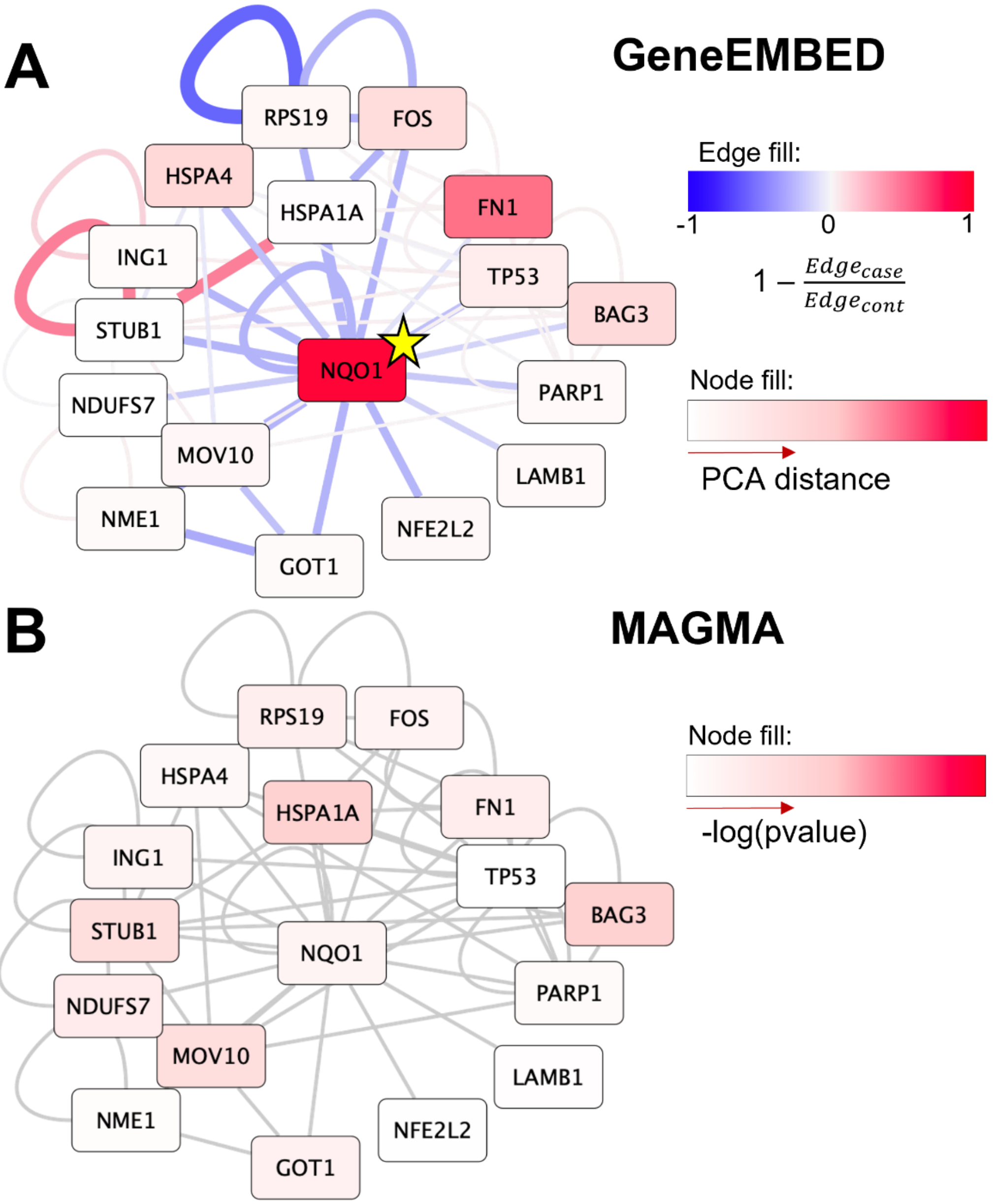

Figure S5. Visual example of GeneEMBED's network informed gene discovery, Related to Figure 1. (A) Network of *NQO1* from the Brain network. Edge color represents the zero-centered ratio of mutation edge weight in cases versus controls. Edge width represents the magnitude of this ratio. Node fill is represented by PCA distance from GeneEMBED on the Discovery cohort using EA. The star on *NQO1* indicates that this gene was identified with FDR < 0.01 in GeneEMBED analysis. (B) shows the same network but with node fill corresponding to the -log(pvalue) from MAGMA analysis on the Discovery cohort. Subtle network differences allow GeneEMBED to identify *NQO1* when mutational data alone would not suffice.

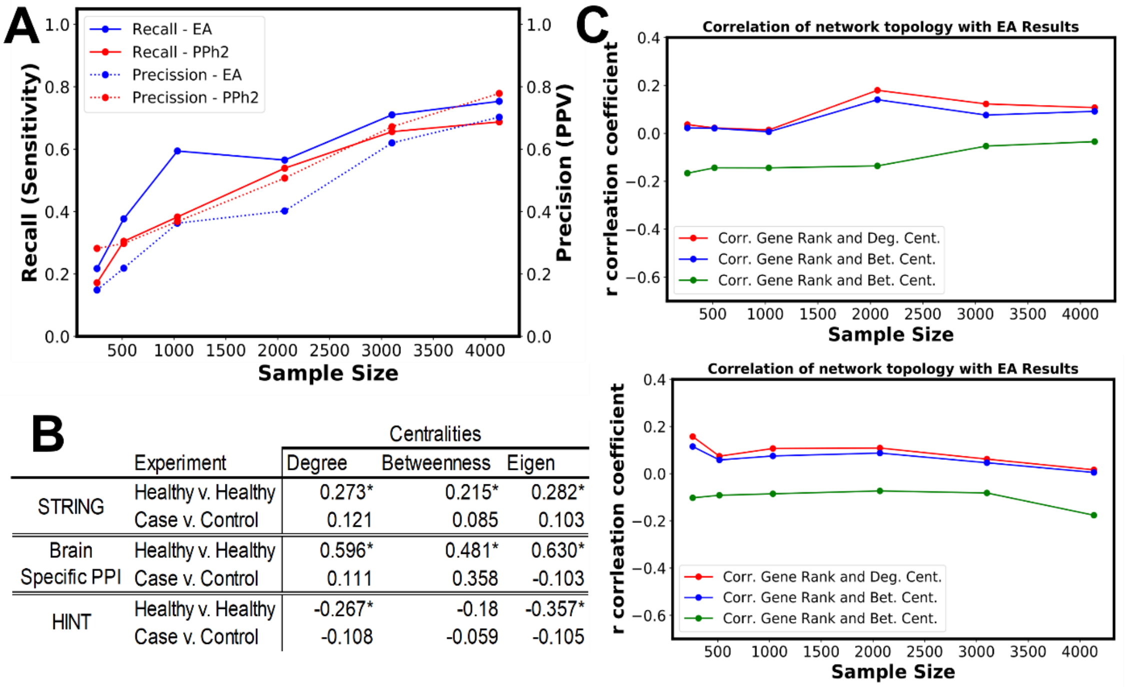

	Experiment	Centralities: Degree	Betweenness	Eigen
STRING	Healthy v. Healthy	0.273*	0.215*	0.282*
	Case v. Control	0.121	0.085	0.103
Brain Specific PPI	Healthy v. Healthy	0.596*	0.481*	0.630*
	Case v. Control	0.111	0.358	-0.103
HINT	Healthy v. Healthy	-0.267*	-0.18	-0.357*
	Case v. Control	-0.108	-0.059	-0.105

Figure S6. GeneEMBED is robust to low sample sizes, Related to Figure 1. (A) Plot of precision and recall of GeneEMBED identified genes at decreased sample sizes relative to genes identified using the full Discovery cohort. (B) Spearman rank-order correlation between genes identified using the three brain networks applied to Healthy vs Healthy controls or case vs control experiment. Asterisk indicates statistically significant ($p<0.05$) correlation. When disease relevant information is removed from data, GeneEMBED relies on network topology to rank genes. (C) Spearman rank-order correlation between candidates identified at low cohort sizes.

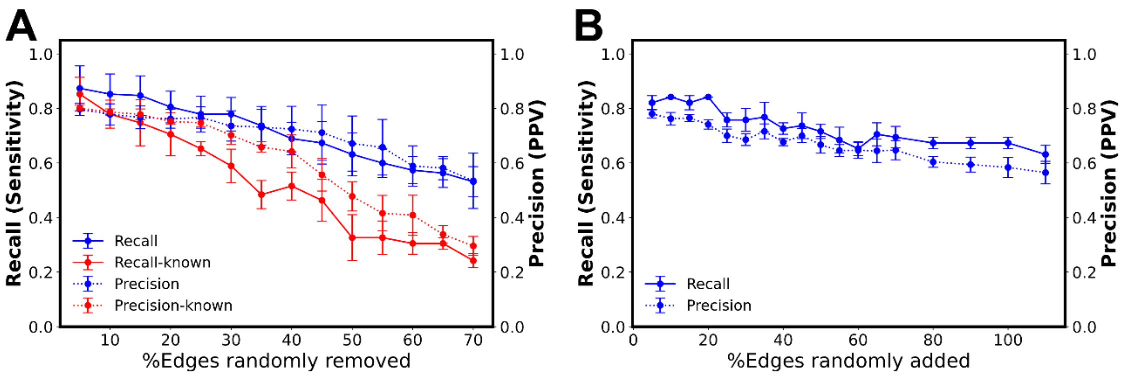

Figure S7. GeneEMBED is robust to false negative and false positive edges, Related to Figure 1. (A) Edges were synthetically and randomly deleted from the Brain network to test sensitivity of GeneEMBED to false negative edges. In blue are plots of precision and recall of GeneEMBED identified genes at various levels of randomly deleted edges. In red are plots of precision and recall of GeneEMBED identified genes when randomly deleted edges are targeted for known (previously identified) genes. (B) Edges were synthetically and randomly added to the Brain network to test sensitivity of GeneEMBED to false positive edges. The plot shows precision and recall of GeneEMBED identified genes at various levels of synthetically added edges. X-axis of '% Edges Added' is relative to the original network size, e.g. at 100%, ~48k edges are randomly added.
